# Platelet count on preoperative day 1 predicts the long-term responses to laparoscopic splenectomy for Chinese patients with medically refractory idiopathic thrombocytopenic purpura

**DOI:** 10.1186/s12893-018-0446-8

**Published:** 2018-11-26

**Authors:** Rui Liao, Pei-Yuan Tang, Jun-Feng Song, Ke-Le Qin, Xun Wang, Xiong Yan

**Affiliations:** Department of Hepatobiliary Surgery, The First Affiliated Hospital of Chongqing Medical University, No.1 Youyi Road, Chongqing, 400016 China

**Keywords:** Idiopathic thrombocytopenic purpura, Laparoscopic splenectomy, Platelet count, Response, Prognosis

## Abstract

**Background:**

Laparoscopic splenectomy (LS) is regarded as a second-line treatment for medically refractory idiopathic thrombocytopenic purpura (ITP), but the predictive factors for the long-term postoperative responses to ITP are still a matter of debate. We aimed to investigate the factors that can predict the long-term response after LS for Chinese patients with medically refractory ITP.

**Methods:**

From January 2011 to September 2016, 78 Chinese patients with ITP who underwent LS were retrospectively analyzed. Twelve parameters were analyzed by univariate and multivariate methods.

**Results:**

Univariate analysis revealed that platelet count on preoperative day (PRD) 1 (*P* < 0.001) and operative time (*P* = 0.011) were significantly associated with long-term response of ITP after LS. Multivariate analysis revealed that platelet count on PRD 1 was a predictive factor of long-term response (P < 0.001). Furthermore, a long-term, stable response of platelet count on PRD 1 of > 30.0 × 10^9^/L was easier to achieve than a platelet count on PRD 1 ≤ 30.0 × 10^9^/L after LS for ITP.

**Conclusions:**

LS is a valuable and effective option in the treatment of medically refractory ITP. Platelet count on PRD 1 is an independent predicting factor for long-term response after LS for Chinese patients with ITP.

## Background

Idiopathic thrombocytopenic purpura (ITP) is a complicated hematologic disorder with a low platelet count, which is characterized by the autoimmune-mediated reduction of platelet production. The first-line treatment option consists of medical therapy with steroids and other immunomodulatory agents [1]. Recently, several platelet growth factors such as eltrombopag and romiplostim are available for ITP treatment [2–4]. Generally, laparoscopic splenectomy (LS) is regarded as a second-line treatment for refractory ITP or the unacceptable medication toxicity, which provides the possible cure for ITP with minimally invasive advantages over open surgery. Hence, LS is the current gold-standard surgical treatment for ITP [5], and the total remission rate can reach 70–90%. [6, 7]

Of note, splenectomy renders patients vulnerable to opportunistic infection such as life-threatening, overwhelming post-splenectomy infection [7]. LS-ineffective ITP patients lose their organs and are no longer eligible for oral tolerance therapy. [8] Therefore, prediction of the efficacy of LS for ITP is important. However, numerous clinical studies on the predictive factors such as age, [9] disease course, [10] accessory spleen removal, [11] platelet counts, [12, 13] response to steroids, [13] and so on have been published, but results are still a matter of debate among surgeons because of a lack of consensus on standardized definitions, which results in these factors not being consistently confirmed. For example, several clinical studies reported that only a high platelet count on postoperative day 7 [6] or 3 months [12] could predict a good response to splenectomy, but a retrospective cohort study showed that perioperative platelet counts are also predictive factors of long-term response after LS for medically refractory ITP. [14] For pediatric patients with chronic ITP, lower preoperative platelet count and postoperative platelet count after one month are associated with relapse after LS. [15] Given these results, it is imperative for the response and outcome criteria to be based on standardized definitions that are adopted to provide accurate reporting and comparisons rather than on individual opinions. Following the definitions and response criteria as stated by the American Society of Hematology 2011 evidence-based practice guideline, [8] we retrospectively analyzed the clinical data of Chinese ITP patients undergoing LS in our department, hoping to find some clues and thereby identify possible predictive response factors and avoid unnecessary surgery for LS-ineffective ITP patients.

## Methods

### Patient population

The clinical data of 87 Chinese patients with ITP who underwent LS in the Department of Hepatobiliary surgery of the First Affiliated Hospital of Chongqing Medical University from January 2011 to September 2016 were collected. The including criteria were (1) ITP cases finally diagnosed through bone marrow aspiration, excluding thrombocytopenia or other causes; (2) treatment with corticosteroid and intravenous immunoglobulin that proved to be insignificant, ineffective, or intolerable; (3) operations completed by the same surgical team; and (4) all underwent total laparoscopic splenectomy. The exclusion criteria were (1) cases with incomplete clinical or follow-up data; (2) cases converted to laparotomy in operation. Three cases for the first reason and six cases for the second were excluded. Finally, 78 cases were included in this study (Table 1). Usually, preoperative imaging such as abdomen ultrasonography, abdominal CT or MRI examination was performed to detect the size of spleens, accessory spleens and so on. Informed consent to use their data in research obtained from all participants was written before surgery. This study was approved by the Ethics Review Committee of the First Affiliated Hospital of Chongqing Medical University.

**Table 1:** Characteristics of Patients

Factors	Overall (*n* = 78)	CR + R (*n* = 65)	NR (*n* = 13)	*P* ^*^
Gender (Female/Male)	44/34 (56.4%/43.6%)	36/29 (55.4%/44.6%)	8/5 (61.5%/38.5%)	0.683
Age, yr., median, (range)	43.8 (14.2~ 77.5)	42.7 (14.2–69.0)	49.4 (35.0–77.5)	**0.013**
Duration of disease, yr., median (range)	12 (6–132)	18 (6–78)	4 (1–132)	0.370
Response to corticosteroid (yes:no)	58:20	50:15	8:5	0.246
PLT count, 10^9^/L, median (range)				
PRD 1	44.0 (4.0–394.0)	47.0 (22.0–394.0)	21.0 (4.0–138.0)	**0.001**
POD 1	93.0 (21.0–415.0)	94.0 (59–415.0)	88.0 (21.0–156.0)	0.681
POD 3	195.0 (112.0–422.0)	198.0 (112.0–422.0)	188.0 (123.0–415.0)	0.503
POD 7	250.0 (189.0–592.0)	250.0 (189.0–592.0)	244.0 (191.0–514.0)	0.489
Operative time, minutes, median (range)	146.0 (65.0~ 280.0)	165.0 (65.0–201.0)	139.0 (110.0–189.0)	0.305
Intraoperative blood loss, ml, median (range)	200 (10–1200)	200 (10–650)	280 (50–1200)	**0.006**
Accessory spleen (yes: no)	6:72	6:59	0:13	0.583
Peroerpative complications (yes:no)	7:71	5:60	2:11	0.056

Abbreviations: CR: complete response; R: response; NR: no response; PLT: platelet; PRD: preoperative day; POD: postoperative day

^*^P value comparison: CR + R vs NR. Data in bold represents *P* < 0.05

Primary ITP was defined according to the American Society of Hematology 2011 evidence-based practice guideline as a platelet count less than 100 × 10^9^/L without other causes or disorders that may be associated with thrombocytopenia. [8] Responses were defined as follows: complete response (CR): a platelet count ≥100 × 10^9^/L and the absence of bleeding; response (R): a platelet count ≥30 × 10^9^/L but < 100 × 10^9^/L and a doubling from baseline and the absence of bleeding; no response (NR): a platelet count < 30 × 10^9^/L or a less than two-fold increase in platelet count from baseline or the presence of bleeding. [8] The blood specimen was extracted from the ulnar vein from patients.

### Surgical procedure

All procedures were performed by the same staff surgeon (Dr. Xiong Yan) with two surgical residents, an experienced laparoscopic surgical team that had completed more than 100 cases of LS. Briefly, LS was performed in a four-trocar technique. After carefully exploring for accessory spleens, the colosplenic, gastrosplenic, and splenophrenical ligaments were dissected with an ultrasonic dissector or a vessel-sealing system in accordance to the hanging-spleen maneuver. The splenic pedicle was transected en bloc with the aid of a 45-mm linear laparoscopic stapler. After mobilization, the spleen was captured and morcellated in an extraction bag. As an indicator for postoperative bleeding or pancreatic fistula, a drainage tube was placed in the upper left quadrant in all patients routinely.

### Follow-up

All patients had a regular follow-up for six to 56 months, and written informed consents were obtained. After surgery and discharge, thrombocyte count (at an interval of at least 7 days), corticosteroid use, mucocutaneous hemorrhage, and visceral hemorrhage events were recorded. Hallmarks for follow-up were sex, age, course of disease (from diagnosis to surgery), perioperative platelet count (PRD 1 and postoperative day [POD] 1, 3, and 7), preoperative response to corticosteroid (effective, dependent, or ineffective), preoperative complications, accessory spleen, operative time, and intraoperative bleeding volume.

### Statistical analysis

For continuous variables with normal distribution, data are expressed as mean ± standard deviation (^−^X ± S) for continuous variables with skewed distribution, median, and range. Categorical variables were compared using the *χ*^2^ test or Fisher’s exact test. Continuous variables were compared using Student’s *t*-test or non-parametric Mann-Whitney U-tests. Univariate analysis and multivariate Cox proportional hazards models were used to estimate the predictors of response after LS for ITP. All analyses were two-sided test and considered of statistical significance when P <0.05. The data were processed by SPSS v.19.0.

### Results

#### Baseline characteristics

The baseline characteristics of 78 Chinese ITP patients after LS are described in Table 1. Among them, there were 34 men and 44 women with a median age of 43.8 years (range, 14.2–77.5 years). The median duration of disease was 12.0 months (range, 6.0–132.0 months). Of the 78 incidents of refractory ITP cases, ten (12.8%) patients had associated diseases, including 3 with diabetes, 3 with viral hepatitis type B, 1 with cholecystolithiasis, 1 with hypertension, 1 with Brenner tumor, and 1 with hypoproteinemia, respectively. The median platelet count on PRD 1 was 44.0 × 10^9^/L (range, 4.0–394.0 × 10^9^/L).

### Operative outcome

Neither postoperative death nor subsequent fatal infections were observed. The median operation time was 146 min (range, 65.0~ 280.2 min), and median intraoperative blood loss was around 200 ml (range, 10–1200 ml). Single or multiple accessory spleens were found in 6 patients (7.7%). No malignancy or other specific finding was confirmed by pathology. Complications related to LS occurred in 7 of 78 patients (9.0%), including five patients with postoperative bleeding, one patient with abdominal abscess, and one patient with wound infection, respectively. Patients’ oral intake began quickly after LS. The median postoperative hospitalization was 6 days (range 3–18 days).

### Platelet response

**Fig. 1:**
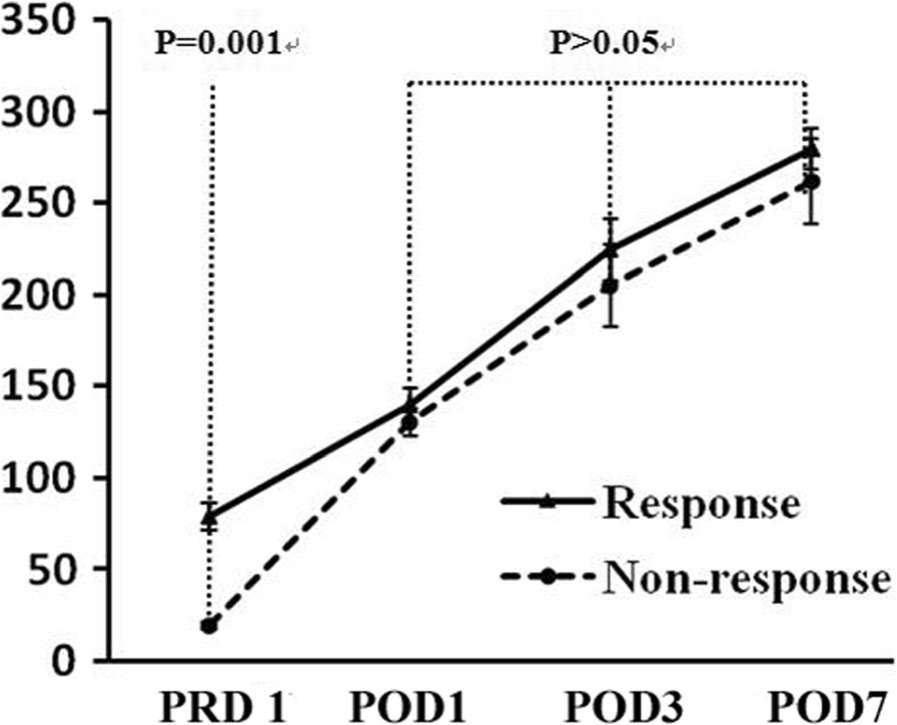
Platelet count on PRD 1 in Group CR + R was significantly higher than those in Group NR (*P* < 0.001). Platelet counts on POD 1, 3 and 7 had no significant difference between two groups (*P* > 0.05). Data are presented as means±SD. Abbreviations: PRD: preoperative day; CR: complete response; R: response; NR: no response; POD: postoperative day

The platelet response to LS was monitored regularly. On the whole, the median platelet counts were elevated promptly from 44.0 × 10^9^/L (range, 4.0–394.0 × 10^9^/L) on PRD 1 to 250.0 × 10^9^/L (range, 189.0–592.0 × 10^9^/L) on POD 7 after surgery (Table 1 and Fig. 1). Taking patients with CR and R together, a total of 71 of 78 (91.0%) patients responded from LS, observed immediately after the operation, and 7 of 78 (9.0%) patients failed to respond. After a median follow-up of 18 months (range, 6–56 months), among the 71 patients with initial CR and R, 6 patients had a loss of response or relapse during the follow-up period. Finally, with regard to the long-term response, a total of 65 of 78 (83.3%) patients had stable remission and no need for further therapy for ITP after LS. Out of 65 patients with long-term responses, 60 patients had a CR, and 5 patients had an R at the last follow-up.

There were no significant differences between the two groups in gender, disease duration, response to corticosteroid, accessory spleen presence, preoperative complications, and postoperative platelet count (Table 1). Compared with those in Groups CR and R, patients in Group NR were older (*P* = 0.013), they had significantly lower preoperative platelet count (*P* = 0.001) and more intraoperative blood loss (*P* = 0.006) before surgery.

### Predictive factors for CR and R

**Fig. 2:**
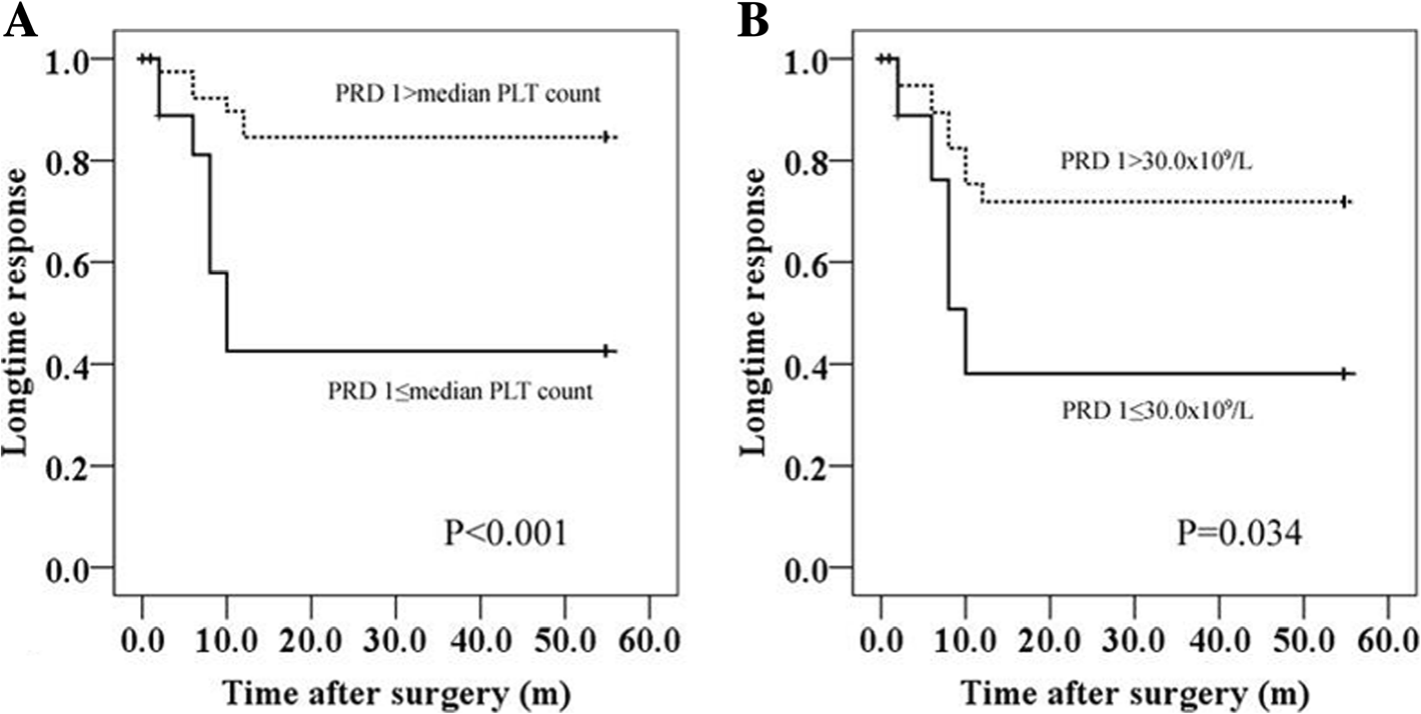
Platelet count on PRD 1 predicts longtime response for ITP after LS during the long-term follow up when the median was used as cut-off value (P < 0.001, A) or the platelet count was divided into two groups: > 30.0 × 10^9^/L and ≤ 30.0 × 10^9^/L (*P* = 0.034, B). Abbreviations: PRD: preoperative day

All data of patient characteristics and surgical results were used for univariate analysis in association with the achievement of response after the medians were used as cut-off values (Table 2). Platelet count on PRD 1 and operative time were demonstrated to be related to long-term response for ITP after LS (*P* < 0.001 and *P* = 0.011, respectively). Then, multivariate analyses were used to examine the association between significant clinical factors and long-term response. Platelet count on PRD 1 (P < 0.001, Fig. 2A) other than operative time (*P* = 0.051) proved to be an independent predictive factor for CR and R for ITP after LS.

**Table 2:** Independent Risk Factors Predicting the longtime response for ITP after LS

Factors	Univariate analysis		Multivariate analysis	
	HR (95%CI)	P-value	HR (95%CI)	*P*-value
Age (years)	_	0.194	_	NA
Gender (Female/Male)	_	0.683	_	NA
Duration of disease	_	0.094	_	NA
PLT count on PRD 1	1.329 (0.851–1.807)	< 0.001*	0.932~ 0.997	< 0.001*
PLT count on POD 1	_	0.087	_	NA
PLT count on POD 3	_	0.174	_	NA
PLT count on POD 7	_	0.065	_	NA
Operative time,	1.573 (0.852–2.294)	0.011*	0.967~ 1.000	0.051
Intraoperative bleeding volume, ml,	_	0.220	_	NA
Accessory spleen (yes: no)	_	0.569	_	NA
Response to hormone (yes:no)	_	0.417	_	NA
Peroerpative complications (yes:no)	_	0.096	_	NA

Abbreviations: ITP: immune thrombocytopenic purpura; LS: laparoscopic splenectomy; PLT: platelet; PRD: preoperative day; POD: postoperative day; *P < 0.05

In this study, the median platelet counts on PRD 1 were 47.0 × 10^9^/L and 21.0 × 10^9^/L in Groups CR + R and Group NR, respectively, which was consistent with a previous report stating that patients with preoperative platelet counts < 40.0 × 10^9^/mL had poor response to steroid or IgG boosting. [14] Here, we further analyzed whether a lower preoperative platelet count such as ≤30.0 × 10^9^/L could predict poor response to ITP after LS because low platelet count (≤30.0 × 10^9^/L) is considered less beneficial in LS with higher morbidity and a low chance to achieve stable response. [14, 16] Our data showed that platelet count on PRD 1 > 30.0 × 10^9^/L made it easier to achieve a long-term stable response than platelet count on PRD 1 ≤ 30.0 × 10^9^/L after LS for ITP (*P* = 0.034, Fig. 2B).

## Discussion

Although LS is the second-line therapy for refractory ITP, it is a proper intervention and the most effective treatment, superior to various medical therapies, and yields long-lasting responses (CR and R) in most patients. [5, 17] The present study showed that 83.3% (65/78) refractory ITP patients achieved a long-term response after LS during our follow-up periods; thus, 16.7% of all patients had no response. The site of platelet destruction from autoantibodies binds to platelets, or antiplatelet-specific plasma cells are related to the poor response. [18–20] However, it is difficult for surgeons to explore the site of platelet destruction. Therefore, it is important to evaluate the adverse factors response for the outcomes of ITP after LS. Moreover, we reevaluated some potential perioperative predictors based on the consensus criteria as defined in 2011 and examined whether they had the same predictive value as reported in older studies.

Here, our long-term observations revealed that platelet count on PRD 1, rather than postoperative platelet count, was associated with a long-term response after LS for medically refractory ITP. This result is supported by Rijcken’s research [14], reporting that patients with high preoperative platelet count boosted with steroids and immunoglobulins had a stable long-term response. Another clinical study, with a mean follow-up of 22 months, also suggested that a higher preoperative platelet count (greater than 70 × 10^9^/L) could predict a successful response to LS. [21] In terms of safety, keeping a higher preoperative platelet count is no doubt an appropriate surgical strategy for a safer operation and a benefit to postoperative recovery. [22] Moreover, few studies had an accurate definition for “preoperative day.” From admission to the day of surgery, platelet count could change significantly, especially after boosting with corticosteroid and immunoglobulins, receiving platelet transfusion, or/and persistent platelet destruction in the spleen, which might result in different outcomes from surgery. Interestingly, our data demonstrated that platelet count on PRD 1 higher than 30.0 × 10^9^/L had a good response to LS for medically refractory ITP. First, a lower preoperative platelet count might reflect not only platelet destruction in a high splenic capacity but also insufficient platelet production contributed to by incomplete megakaryopoiesis to functional platelets in specific bone marrow niches that would perhaps result in NR after splenectomy. [23, 24] Second, compared to other time points before surgery, platelet count on PRD 1 seems to reflect an authentic milieu in the spleen more accurately, which is closely related to preoperative platelet destruction. Third, from the clinical point of view, a platelet count of at least 30–50 × 10^9^/L is supposed to be the “safe” level by most surgeons. A low preoperative platelet count of ≤30 × 10^9^/L is proposed to be less beneficial in LS, with more platelet transfusion requirements, and about one-third of transfused platelets are sequestered in the splenic pool and destroyed. [25, 26] Thus, a combination of reducing platelet destruction and stimulating platelet production may increase the efficacy and chances of a favorable outcome in ITP. The patient with low platelet count receiving preoperatively another course of steroids or IgG or eltrombopag or romiplostim maybe should tried out, which could be validated by prospective trials. The optimal perioperative management, especially for patients with ultra low PLT should be developed, such as hematologist’s assessment for potential treatment strategy, preoperative platelet transfusions preparation, intraoperative platelet transfusions, dissecting and clipping the splenic artery as early in the course of the operation as possible [27].

Although some literature is available mentioning other important predictive factors such as postoperative platelet count, age, response to hormone, and disease course, [6, 12, 14, 28] current results showed only that low platelet count on PRD 1 was an independent postoperative response predictor for ITP. Several aspects may contribute to this discrepancy. The first may be different sample sizes and patient characteristics. The second reason could be the heterogeneous nature of ITP. Furthermore, different including criteria and definitions of ITP and response would lead to these inconsistencies. Finally, various discontinuations of medical therapy such as corticosteroids, immunoglobulins, or rituximab should also be taken into account. However, these factors are not the decisive surgical indications, because patients without these predictors still have a reasonable chance of response. Actually, long-term response after LS is crucial for patients with medically refractory ITP.

The present study supported splenectomy as a safe management option with a low complication rate (9.0%) and intraoperative blood loss (median 200 ml in overall), and no deaths were recorded. For the treatment of ITP, LS is a less complicated procedure for ITP, with a relatively short operative time (median 146 min overall). However, it is still noteworthy that the risk of overwhelming sepsis and thrombosis remain long-term threats for ITP patients after LS.

The present study has several limitations. The first is the retrospective nature of the design. Moreover, this was a single-center study in China with a small sample size. In the future, we need to investigate more preoperative factors, including platelet count on various preoperative days. Hence, these limitations precluded us from drawing any firm conclusions. Larger-size, multicenter, and prospective trials are merited to validate our findings.

## Conclusions

In summary, LS is a valuable and effective option in the treatment of medically refractory ITP because the majority of patients (65/78, 83.3%) achieved long-term responses in this study. Moreover, a preoperative 1-day platelet count is an independent predictive factor for surgical efficacy in these Chinese patients. Furthermore, uniform definitions of response obtained some similar results to former surgical studies. Additional studies are warranted to identify predictive factors of response to surgical therapy and might facilitate optimization of the selection of patients suitable for surgery.

## Abbreviations

CR: complete response
ITP: idiopathic thrombocytopenic purpura
LC: Laparoscopic splenectomy
NR: no response
POD: postoperative day
PRD: preoperative day
R: response

## References

[1] Cooper N (2017). State of the art - how I manage immune thrombocytopenia. Br J Haematol.

[2] Zhang J, Liang Y, Ai Y, Li X, Xie J, Li Y, Zheng W, He R (2018). Eltrombopag versus romiplostim in treatment of adult patients with immune thrombocytopenia: a systematic review incorporating an indirect-comparison meta-analysis. PLoS One.

[3] Grainger JD, Locatelli F, Chotsampancharoen T, Donyush E, Pongtanakul B, Komvilaisak P, Sosothikul D, Drelichman G, Sirachainan N, Holzhauer S (2015). Eltrombopag for children with chronic immune thrombocytopenia (PETIT2): a randomised, multicentre, placebo-controlled trial. Lancet.

[4] Tarantino MD, Bussel JB, Blanchette VS, Despotovic J, Bennett C, Raj A, Williams B, Beam D, Morales J, Rose MJ (2016). Romiplostim in children with immune thrombocytopenia: a phase 3, randomised, double-blind, placebo-controlled study. Lancet.

[5] Chater C, Terriou L, Duhamel A, Launay D, Chambon JP, Pruvot FR, Rogosnitzky M, Zerbib P (2016). Reemergence of splenectomy for ITP second-line treatment?. Ann Surg.

[6] Ojima H, Kato T, Araki K, Okamura K, Manda R, Hirayama I, Hosouchi Y, Nishida Y, Kuwano H (2006). Factors predicting long-term responses to splenectomy in patients with idiopathic thrombocytopenic purpura. World J Surg.

[7] Kang CM, Lee JG, Kim KS, Choi JS, Lee WJ, Kim BR, Ko YW, Han JS, Min YH (2007). Long-term follow-up of laparoscopic splenectomy in patients with immune thrombocytopenic purpura. J Korean Med Sci.

[8] Neunert C, Lim W, Crowther M, Cohen A, Solberg L, Crowther MA (2011). 0The American Society of Hematology 2011 evidence-based practice guideline for immune thrombocytopenia. Blood.

[9] Gonzalez-Porras JR, Escalante F, Pardal E, Sierra M, Garcia-Frade LJ, Redondo S, Arefi M, Aguilar C, Ortega F, de Cabo E (2013). Safety and efficacy of splenectomy in over 65-yrs-old patients with immune thrombocytopenia. Eur J Haematol.

[10] Kojouri K, Vesely SK, Terrell DR, George JN (2004). Splenectomy for adult patients with idiopathic thrombocytopenic purpura: a systematic review to assess long-term platelet count responses, prediction of response. and surgical complications Blood.

[11] Sampath S, Meneghetti AT, MacFarlane JK, Nguyen NH, Benny WB, Panton ON (2007). An 18-year review of open and laparoscopic splenectomy for idiopathic thrombocytopenic purpura. Am J Surg.

[12] Wang M, Zhang M, Zhou J, Wu Z, Zeng K, Peng B, Niu T (2013). Predictive factors associated with long-term effects of laparoscopic splenectomy for chronic immune thrombocytopenia. Int J Hematol.

[13] Aleem A (2011). Durability and factors associated with long term response after splenectomy for primary immune thrombocytopenia (ITP) and outcome of relapsed or refractory patients. Platelets.

[14] Rijcken E, Mees ST, Bisping G, Krueger K, Bruewer M, Senninger N, Mennigen R (2014). Laparoscopic splenectomy for medically refractory immune thrombocytopenia (ITP): a retrospective cohort study on longtime response predicting factors based on consensus criteria. Int J Surg.

[15] Kim DJ, Chung JH (2014). Long-term results of laparoscopic splenectomy in pediatric chronic immune thrombocytopenic purpura. Ann Surg Treat Res.

[16] Keidar A, Sagi B, Szold A (2003). Laparoscopic splenectomy for immune thrombocytopenic purpura in patients with severe refractory thrombocytopenia. Pathophysiol Haemost Thromb.

[17] Zheng CX, Zheng D, Chen LH, Yu JF (2011). Wu ZM: laparoscopic splenectomy for immune thrombocytopenic purpura at a teaching institution. Chin Med J.

[18] Thachil J (2014). Alternate considerations for current concepts in ITP. Hematology.

[19] McMillan R (2000). The pathogenesis of chronic immune (idiopathic) thrombocytopenic purpura. Semin Hematol.

[20] Mahevas M, Patin P, Huetz F, Descatoire M, Cagnard N, Bole-Feysot C, Le Gallou S, Khellaf M, Fain O, Boutboul D (2013). B cell depletion in immune thrombocytopenia reveals splenic long-lived plasma cells. J Clin Invest.

[21] Duperier T, Brody F, Felsher J, Walsh RM, Rosen M, Ponsky J (2004). Predictive factors for successful laparoscopic splenectomy in patients with immune thrombocytopenic purpura. Arch Surg.

[22] Provan D, Stasi R, Newland AC, Blanchette VS, Bolton-Maggs P, Bussel JB, Chong BH, Cines DB, Gernsheimer TB, Godeau B (2010). International consensus report on the investigation and management of primary immune thrombocytopenia. Blood.

[23] Khodadi E, Asnafi AA, Shahrabi S, Shahjahani M, Saki N (2016). Bone marrow niche in immune thrombocytopenia: a focus on megakaryopoiesis. Ann Hematol.

[24] Arnold DM, Nazi I, Toltl LJ, Ross C, Ivetic N, Smith JW, Liu Y, Kelton JG (2015). Antibody binding to megakaryocytes in vivo in patients with immune thrombocytopenia. Eur J Haematol.

[25] Habermalz B, Sauerland S, Decker G, Delaitre B, Gigot JF, Leandros E, Lechner K, Rhodes M, Silecchia G, Szold A (2008). Laparoscopic splenectomy: the clinical practice guidelines of the European Association for Endoscopic Surgery (EAES). Surg Endosc.

[26] Estcourt LJ, Birchall J, Allard S, Bassey SJ, Hersey P, Kerr JP, Mumford AD, Stanworth SJ, Tinegate H (2017). Guidelines for the use of platelet transfusions. Br J Haematol.

[27] Zychowicz A, Radkowiak D, Lasek A, Malczak P, Witowski J, Major P, Strzalka M, Kulawik J, Budzynski A, Pedziwiatr M (2018). Laparoscopic splenectomy for immune thrombocytopenia in patients with a very low platelet count. Wideochir Inne Tech Maloinwazyjne.

[28] Patel NY, Chilsen AM, Mathiason MA, Kallies KJ, Bottner WA (2012). Outcomes and complications after splenectomy for hematologic disorders. Am J Surg.

